# Deep Mind 21 functional does not extrapolate to transition metal chemistry†

**DOI:** 10.1039/d4cp00878b

**Published:** 2024-04-04

**Authors:** Heng Zhao, Tim Gould, Stefan Vuckovic

**Affiliations:** a Department of Chemistry, University of Fribourg Fribourg Switzerland stefan.vuckovic@unifr.ch; b Queensland Micro- and Nanotechnology Centre, Griffith University Nathan Qld 4111 Australia

## Abstract

The development of density functional approximations stands at a crossroads: while machine-learned functionals show potential to surpass their human-designed counterparts, their extrapolation to unseen chemistry lags behind. Here we assess how well the recent Deep Mind 21 (DM21) machine-learned functional [*Science*, 2021, **374**, 1385–1389], trained on main-group chemistry, extrapolates to transition metal chemistry (TMC). We show that DM21 demonstrates comparable or occasionally superior accuracy to B3LYP for TMC, but consistently struggles with achieving self-consistent field convergence for TMC molecules. We also compare main-group and TMC machine-learning DM21 features to shed light on DM21's challenges in TMC. We finally propose strategies to overcome limitations in the extrapolative capabilities of machine-learned functionals in TMC.

## Abstract 2

The development of density functional approximations stands at a crossroads: while machine-learned functionals show potential to surpass their human-designed counterparts, their extrapolation to unseen chemistry lags behind.

## Graphical abstract

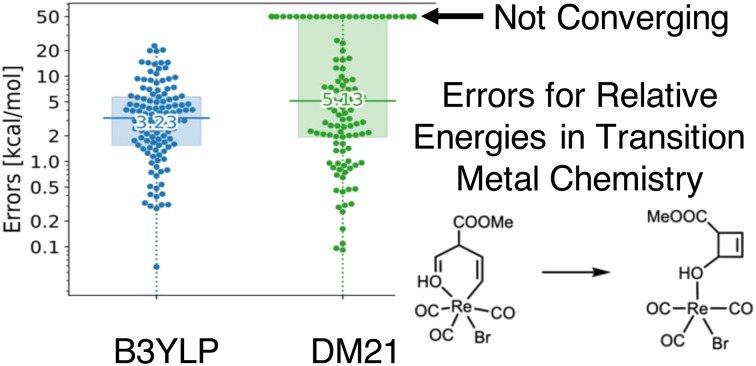

## I. Introduction

The accuracy of density functional approximations (DFAs) has become a limiting factor in scientific discoveries driven by electronic structure calculations and empowered by artificial intelligence.^1–5^ At the same time, the development of DFAs is currently in “no man's land”. On the one hand, machine-learned DFAs hold promise to overcome the known deficiencies of human-designed functionals.^6–13^ Yet, their transferability^14^ remains a major challenge, essential for the broad applicability seen in their human-designed counterparts, such as PBE^15^ or B3LYP.^16–19^

A major step forward in machine learning of accurate DFAs has been achieved by the development of the Deep Mind 21 (DM21) functional.^8^ From the point of view of DFA's classification, DM21 is a machine-learned local hybrid (see ref. 22 for a review of local hybrids and ref. 23 for a recent comparison between human-designed local hybrids and DM21). With the inclusion of fractional charges (FC) and fractional spin (FS) data in the training, DM21 has addressed some of the long-standing deficiencies of standard DFAs linked to their improper behavior for systems with FC and FS.^24^ However, the training of DM21 excludes elements heavier than Krypton, posing questions about its performance in transition metal chemistry (TMC), a realm generally challenging for quantum chemistry due to strong correlation effects and a large number of multireference cases.^20,25–27^

Trained on fractional spin (FS) DM21 can capture some multi-reference effects in main group chemistry, such as stretching covalent bonds, though it encounters difficulties at intermediate bond distances. For example, training DM21 on the hydrogen atom with zero polarization ensures the accurate H_2_ dissociation limit without breaking spin symmetry. Focusing on dimers, main-group dimers primarily exhibit multireference effects when their bonds are stretched, whereas transition metal dimers display these effects even at their equilibrium geometries. Thus, the difference in the nature of multireference effects between main-group and TMC raises the question of whether DM21's ability to capture such effects in the former can extend to the latter. But, given the known shortcomings of standard functionals like B3LYP in describing multireference transition metals (TM), such as TM dimers, even a far less stringent question arises: Does DM21, which was pretrained on B3LYP densities, perform at least not much worse in this domain than B3LYP itself?

**Fig. 1:**
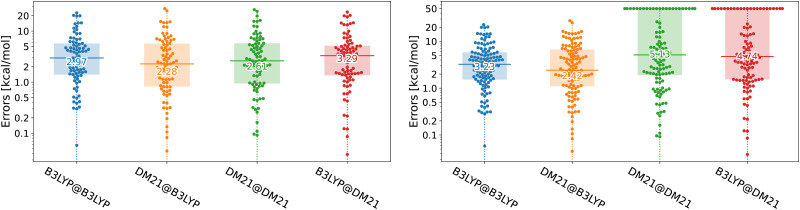
Beeswarm plots with errors for B3LYP and DM21 functionals across TMC117 dataset variations (see the text for the dataset description). The number at the horizontal bar denotes the median absolute error. The TMC117 dataset built from the TMC151 dataset^20^ of Chan and co-workers by excluding large systems where DM21 calculations were prohibitively resource-intensive. The left plot shows 83 reactions which we could converge with DM21, whereas the right shows all 117 reactions, where we set 50 kcal mol^−1^ errors to non-converged cases. *A*@*B* denotes functional *A*'s evaluation on densities/orbitals from functional *B*'s Kohn–Sham calculation. The D3(BJ) dispersion correction^21^ has been applied to all energies.

Unfortunately, in this paper, we show that the answers to both questions regarding DM21's performance in TMC are negative. While DM21, once it converges, yields accuracy for transition metal compounds comparable (in some cases even superior) to B3LYP, it consistently struggles with SCF convergence. We illustrate the performance of DM21 for TMC in Fig. 1 with beeswarm plots showing errors of B3LYP and DM21 functionals (see the caption of the figure for details). The left panel of Fig. 1 shows DM21's potential to surpass B3LYP in TMC. The data indicate a decrease in median error from 3 kcal mol^−1^ for self-consistent B3LYP calculations to 2.3 kcal mol^−1^ when DM21 is applied to B3LYP orbitals. Self-consistent DM21 calculations are in between the two in terms of accuracy with the median error of 2.6 kcal mol^−1^ (other error metrics will follow later). The right panel of Fig. 1 gives a more critical assessment of DM21 for TMC as it includes systems that failed to converge with this functional. For these cases, we (arbitrarily) set errors of 50 kcal mol^−1^, a number reflecting the expected upper limit of DFT errors for the considered TMC reactions. When all reactions are considered in the right panel, DM21 evaluated on B3LYP densities remains accurate; however, roughly 30% of the reactions do not reach SCF convergence under DM21. The major convergence issues with DM21 not only limit its practical applicability for TMC but could also render its use impossible in this area.

As we will show later in the paper, these convergence issues of DM21 cannot be resolved by standard SCF setting adjustments. We demonstrate this by going beyond an SCF procedure and employing a direct orbital optimization algorithm for DM21 cases that could not converge with our SCF protocol. Even then, the DM21 convergence still fails, underscoring a fundamental limitation in DM21's ability to extrapolate to transition metals.

In addition to testing DM21's accuracy for TMCs, we analyze SCF convergence failures for specific TMC systems, compare DFT features of TM molecules against their main-group counterparts (*e.g.*, CrO *vs.* CaO/CO), and demonstrate that the former can be easily missed when training machine-learned functionals.

The paper is organized as follows, computational details are outlined in Section II, followed by Section III with the key numerical and convergence results, Section IV with the analysis of DFT features. Finally, Section V is devoted to conlcusions and outlook.

## II. Computational details

### A. Computational setup

All DFT calculations in this work have been obtained in PySCF.^28^ We use the TMC151^20^ transition-metal datasets compilation, developed by Chan *et al.*, to assess the accuracy of DM21 in TMC. TMC151 includes the TMD60 dataset,^29^ featuring TM dimer dissociation energies; MOR41, with 41 metal–organic reaction energies;^30^ and TMB50 containing barriers of complexes of second- and third-row transition metals.^20^ The current implementation of DM21 is very costly. For example, a single SCF iteration for *n*-decane on 4 CPU cores with def2-QZVP basis set and resolution of identity (RI) approximations (the original implementation of DM21 in PySCF) takes approximately 7 hours. In contrast, a complete B2PLYP double hybrid^31^ calculation with the same settings, without RI approximations, is completed within 13 minutes with RI on or 3 hours with RI off. Therefore, due to the currently high cost of DM21, we excluded reactions with large systems from MOR41 and TMB50, leading to their TMB40 and MOR17 subsets, respectively. TMD60 was kept as is, leading to the streamlined TMC117 subset of TMC151 (TMB40 + MOR17 + TMD60). For TMD60 calculations, we use the def2-QZVP basis set, while for TMB40 and MOR17 we use the def2-TZVP basis set (with corresponding effective core potentials as in ref. 20 for heavier atoms when applicable).^32^ RI approximations are used with corresponding auxiliary basis sets^33^ to accelerate the calculation.

To better understand DM21's relative accuracy to B3LYP for TMC, in addition to assessing their self-consistent performances, we also test their accuracies using cross-evaluated densities^34^ (DM21@B3LYP and B3LYP@DM21, where A@B denotes an evaluation of a functional A on the electron density computed by functional B). For all calculations, we also include the D3(BJ) dispersion correction with the Becke–Johnson damping function^21^ (the results from the paper without D3(BJ) are given in the ESI†). Since self-consistent DM21 and B3LYP use the same D3(BJ) parameters,^8^ we safely assume that the same parameters could be used for DM21@B3LYP and B3LYP@DM21.

### B. SCF protocol

We establish a self-consistent field (SCF) protocol for achieving system convergence with DM21. Our methodology starts with SCF Strategy A, advancing to Strategy B if convergence is not achieved, and then to Strategy C if necessary. As said, we use PySCF^28^ for all our SCF calculations, and inspired by the Orca's SCF settings,^35^ we use the following set of A to C Strategies:

#### Strategy A

Level shifting is set as 0.25, damping factor is 0.7, direct inversion in the iterative subspace DIIS will start at cycle 12 (some of the settings are similar to NormalConv SCF protocol in Orca).

#### Strategy B

Level shifting is set as 0.25, damping factor is 0.85, DIIS starts at cycle 0 (some of the settings are similar to SlowConv SCF protocol in Orca).

#### Strategy C

Level shifting is set as 0.25, damping factor is 0.92, DIIS starts at cycle 0 (some of the settings are similar to VerySlowConv SCF protocol in Orca).

For cases that don't converge we also (unsuccessfully in all attempts) employ Strategy D. This strategy is fundamentally different from A–C as it involves direct optimization of the energy with respect to orbitals. It may thus, in principal, converge for cases where standard SCF procedures break down. Full details are provided in Appendix A.

Between Strategies A–D we have a set of increasingly difficult, but in principal increasingly robust, ways to converge DFAs even in difficult systems. We are now ready to put these strategies into practice, and see how well DM21 performs. Further computational details for all approaches are given in Appendix B.

## III. Results

### A. Convergence of DM21 for transition metal dimers

Before the detailed analysis of DM21 for the TMC117 dataset, we first focus on the SCF convergence issues for TMCs, which, as we will show, represent the major obstacle to the use of DM21 in TMC applications.

In Table 1, we present the convergence success of different SCF strategies for each system within the TMD60 dataset. As said, we start with the SCF Strategy A and move to B or C only if necessary. From Table 1, we can see that for the TMD60 dataset, which includes 60 dimers and 16 atoms, DM21 SCF convergence was successful for 59 systems (45 dimers/14 atoms) using Strategy A. B managed to converge 2 additional dimers, while C and direct energy optimization with D did not lead to further convergence. In stark contrast, all 152 species in the W4-11 (main-group atomization energies)^36^ dataset converged under Strategy A, likely reflecting the use of main-group atomization energies in DM21's training. At the same time, B3LYP's SCF convergence for TMD species was far easier, with almost all directly converging using A and the remaining five *via* B. We can also see from Table 1 that species with V and Cr atoms were particularly difficult for SCF convergence, where only the VO dimer and the Cr atom converged. We note that the use of smaller basis set than def2-QZVP, which we use for TMD60, can lead to the convergence of a few additional species (*e.g.*, within Strategy D and the cc-pVDZ basis set, we could also converge the V atom).

**Table 1:** SCF convergence of all TMD60 species using different strategies presented in Section IIB. ‘x’ denotes species that failed to converge under any strategy. A letter A–D indicates the strategy that successfully converged the (di)atom. No system was successfully converged using Strategies C or D

Element	Atom	H	F	Cl	Br	O	S
Sc	A	A	A	A	A	A	A
Ti	A	A	A	A	A	A	A
V	x	x	x	x	x	B	x
Cr	A	x	x	x	x	x	x
Mn	A	A	A	A	x	x	x
Fe	x	A	A	A	A	B	x
Co	A	A	A	A	A	A	A
Ni	A	A	A	A	A	A	A
Cu	A	A	A	A	A	A	A
Zn	A	A	A	A	A	A	A

The failures of Strategies A–C here raise the question whether the problem lies in SCF approach or DM21. This was indeed the reason why we introduced Strategy D, which involves direct optimization of orbitals and thus bypasses SCF entirely. In principle, D can converge any energy functional that is bounded from below; and can bypass issues with orbital (re-)ordering that are usually treated by level shifting. But, in practice, it requires the energy to be sufficiently smooth with respect to variations in the orbitals. That is, the DFA must vary smoothly in its input features since orbital-dependence is inherited from the (*meta*-)densities and energy densities.

Therefore, DM21's failure to converge for some systems using D suggests that the functional is highly non-smooth (*i.e.* nearly discontinuous) for combinations of input features that are ‘close’ enough to the minima to be sampled during optimization. The presence of (near) discontinuities is not surprising in a machine-learned DFA – the exact density functional is very complicated and the DFA needs to capture that complexity by fitting to training data, so will inherit a bias toward its training data. What is surprising is that even simple systems, like TM atoms, can have combinations of features that are outside the training data. Section IV will therefore explore this point in more detail.

**Fig. 2:**
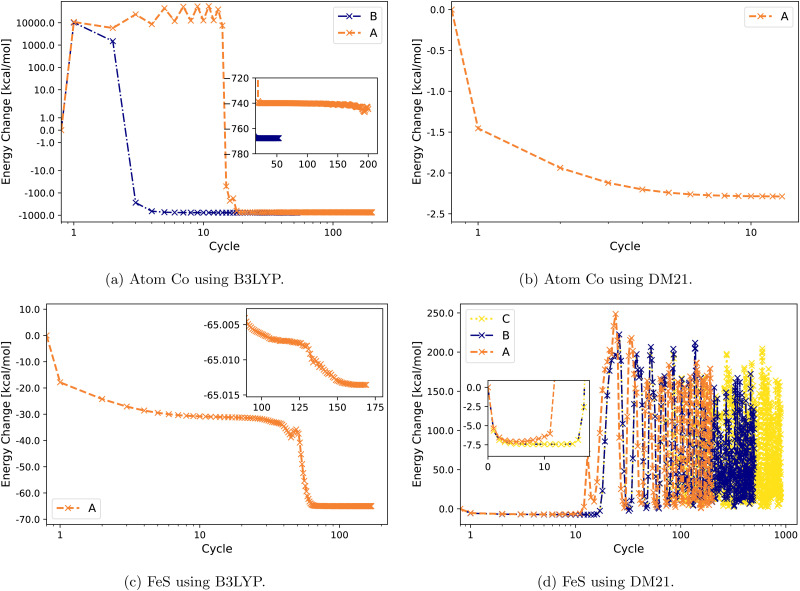
Energy change (zeroed at first iteration) during SCF cycles of Co and FeS with B3LYP and DM21. Note the semi-logarithmic scale in (a).

Fig. 2 illustrates the convergence behavior of Co atom and FeS using B3LYP and DM21. The Co atom converges under B3LYP with Strategies A and B, with a smoother convergence observed using B [Fig. 2(a)]. For the same atom, DM21's SCF convergence initiated with B3LYP-converged orbitals proceeds smoothly with Strategy A. By contrast, for the FeS molecule, DM21 fails to converge with any of the Strategies A, B, or C, as indicated by the erratic energy values with no stabilization even over an extended number of SCF iterations (Fig. 2(d)). However, Fig. 2(c) shows that B3LYP encounters no such convergence issues with FeS.

**Fig. 3:**
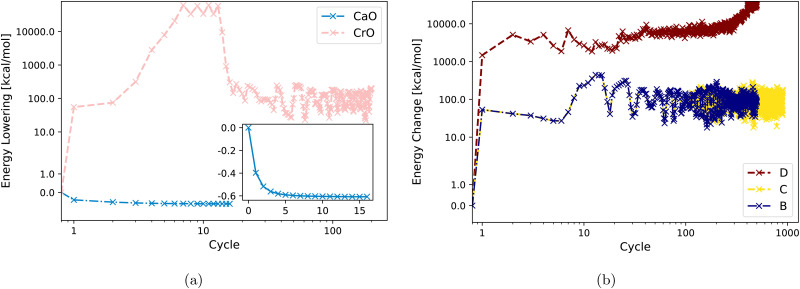
Energy change (zeroed at first iteration) during SCF cycles within DM21 for (a) CaO and CrO using Strategy A. (b) CrO using Strategy B, C, D.

Fig. 3(a) displays DM21 SCF convergence attempts for both CaO and CrO using Strategy A. It shows straightforward convergence for the main-group oxide CaO, whereas the transition metal oxide CrO fails to converge with the same strategy. Fig. 3(b) demonstrates that Strategies B, C, and D are also unsuccessful in achieving SCF convergence for CrO with DM21. Section IV will analyze the input features of CaO and CrO to shed light on their different DM21 SCF convergence behaviors.

The fact that Strategy D [see Fig. 3(b)] tends to increase the energy of CrO is worth commenting on. This behaviour reflects cross-contamination between two numerical issues used in D: (1) the use of an approximate Hessian in Newton iteration for the orbital optimization scheme; (2) non-smoothness of the DM21 DFA as a functional of orbitals. Issue 1 [see eqn (A1) in Appendix A below] can lead the orbital optimization algorithm to sometimes “climb up hills” when the approximate Hessian sometimes has the wrong ‘sign’. In well-behaved systems, or with well-behaved DFAs, the ascent is followed by a descent once the ‘sign’ gets fixed – indeed, ascent sometimes helps the algorithm iterate to the global minima. But Issue 2 (evidenced by very large fluctuations in the energy) makes both the Hessian and its approximation *de facto* discontinuous. Discontinuities can trap the algorithm in regions of orbital space where the energy varies rapidly. Continued iteration may eventually find the minima, although the fluctuations of around 1 *E*_h_ (*i.e.* ∼100 × MAE in atomization energies of converged cases) in CrO certainly make this challenging.

We finally note that the failure to converge using Strategies A–D does not strictly prove that the system cannot be converged (indeed it is unlikely that a minimum does not exist). But, the fact that these systems fail even in Strategy D, which attempts to directly minimize the energy with respect to orbitals, reveals that convergence is extremely difficult.

### B. DM21 performance for TMC117

After analyzing DM21 convergence difficulties in the TMD dataset, Table 2 assesses DM21 across TMC117 datasets: TMD60, TMB40, and MOR17. For the DM21-converged subsets of these datasets, labeled “Sub”, we present mean absolute errors (MAEs) for the following functional combinations: B3LYP@B3LYP, DM21@B3LYP, DM21@DM21, and B3LYP@DM21. For full datasets (“Whole”), only combinations evaluated at B3LYP densities are shown due to convergence issues, highlighting B3LYP@B3LYP and DM21@B3LYP. The table indicates DM21 non-convergence for 34 systems within TMC117 (13 from TMB40 and 21 from TMD60). Given Strategy D's high cost and its inability to converge those TMD60 systems where A–C failed, we did not use it for TMB40 and MOR17 systems. All results in Table 2 include D3(BJ) corrections, with D3(BJ)-free comparisons in Table S-I in the ESI.†

**Table 2:** MAEs (kcal mol^−1^) of different functionals. D3(BJ) correction has been added to all functionals

Dataset	TMB40		TMD60		MOR17
	Whole	Sub	Whole	Sub	Whole
B3LYP@B3LYP	2.43	1.61	6.00	6.41	5.31
DM21@B3LYP	1.62	1.51	6.88	6.25	3.41
DM21@DM21	—	1.81	—	6.59	3.70
B3LYP@DM21	—	1.36	—	6.60	4.86
Number of reactions	40	27	60	39	17

From Table 2, we can see that DM21 has the potential for more accurately describing TMC than B3LYP. For example, we can see that DM21@B3LYP is on average noticeably more accurate than self-consistent B3LYP@B3LYP. While self-consistent DM21@DM21 shows a slight decrease in accuracy compared to DM21@B3LYP, it remains more accurate than B3LYP@B3LYP. In DM21 converged instances, B3LYP@DM21 shows slightly lower but still comparable accuracy to DM21@B3LYP.

The MAEs in Table 2 suggest DM21's potential to outperform B3LYP for TMC both in terms of approximate functional and energetic consequences due to approximate densities. However, a large number of the DM21 unconverged cases in the same table cannot be overlooked. This issue makes DM21 of nearly no use in TMC, as even when DM21 SCF solution is achievable, finding such solution for TMC would require far more human effort and intervention than for *e.g.*, B3LYP.

**Fig. 4:**
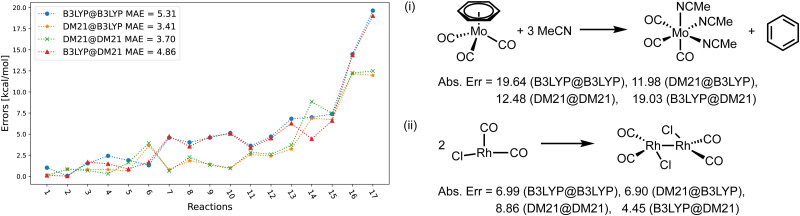
(left) Errors of the four method combinations for MOR17 dataset. def2-TZVP basis set was used and the D3(BJ) correction has been added to all results. (right) Example of reactions in MOR17 with large errors.

**Fig. 5:**
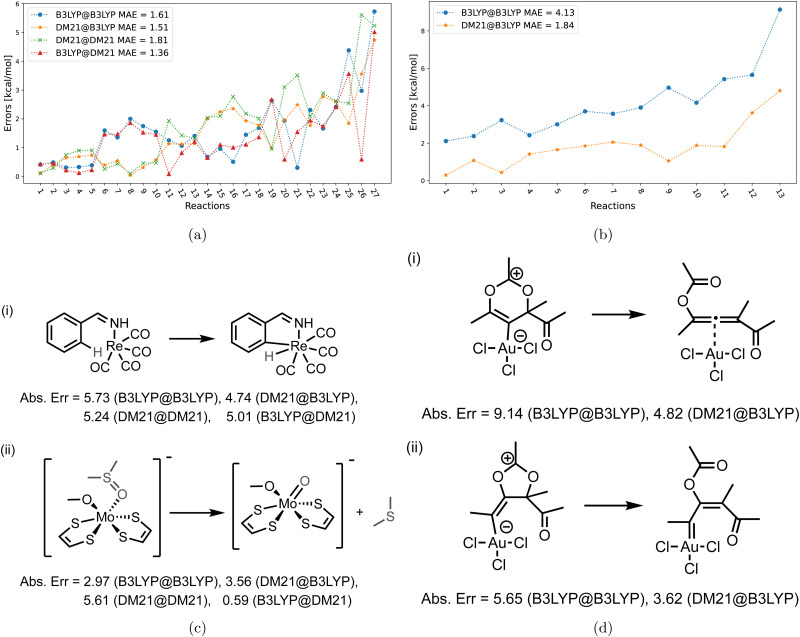
(a) Errors of the four method combinations for TMB40 dataset for a subset of barriers for which DM21 converges. def2-TZVP basis set was used and the D3(BJ) correction has been added to all results. (b) Same as (a) but for barriers for which DM21 did not converge. (c) Examples of reactions from (a) panel with large errors. (d) Examples of reactions from (b) panel with large errors.

**Fig. 6:**
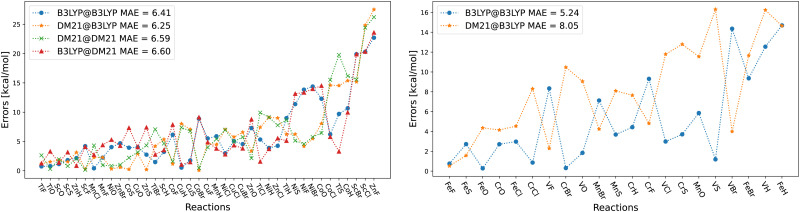
(left) Errors of the four method combinations for TMD60 dataset for a subset of bond energies for which DM21 converges. def2-QZVP basis set was used and the D3(BJ) correction has been added to all results. (right) Same as (left) but for bond energies for which DM21 did not converge.

Fig. 4–6 focus on the performance of the 4 functional/density combinations for the individual reactions of the MOR17, TMB40, and TMD60 sets. Fig. 4 and 5 also contain examples of the most difficult reactions in their sets.

Fig. 4 shows the errors for the MOR17 set, for which we could converge all systems within DM21. We can see that the evaluation of a given functional on the other's density (A@B) is somewhat more accurate than self-consistent calculations (A@A), which is likely due to the error cancellations between functional errors of A and density-driven errors of B.^37–39^ More importantly, we can see that the DM21 functional, whether paired with its own density or that of B3LYP, provides better accuracy for MOR17 than the B3LYP functional.

In Fig. 5, we show the errors for the TMB set, split by the reactions where we could converge the DM21 results [panel (a)], and those where we could not [panel (b)]. From Fig. 5(a), we can see that A@A and A@B curves align for small errors, suggesting that a functional choice determines accuracy. With larger errors, A@A and A@B pairs are less aligned, indicating density's increasing relevance for the energies. Overall, the MAEs of the four DM21/B3LYP methods are smaller than that for MOR17 and lie in a narrow range (1.4–1.8 kcal mol^−1^).

We can see from Fig. 5(b) that for the TMB cases, when DM21 does not converge, DM21@B3LYP is much more accurate than B3LYP@B3LYP. This intriguing improvement of DM21@B3LYP over B3LYP@B3LYP aligns with similar improvements observed for main-group barriers.^8^ On the other hand, this improvement in panel (b) (for TMB40 barriers that did not converge with DM21) is much larger than in panel (a) (cases that converge). This discrepancy suggests a potential trend for TM barriers where DM21 fails to converge, which may be attributed to the error cancellation between DM21's functional error and B3LYP's density-driven errors. However, due to the limited number of such cases, this observation remains speculative.

Fig. 6 focuses on the individual errors for the TMD60 dataset. For cases when DM21 converges [panel (a)], the errors are large and comparable in magnitude across the four methods. In panel (b) with the cases for which DM21 does not converge, DM21@B3LYP performs poorer than B3LYP@B3LYP, which is an opposite trend from Fig. 5. Nevertheless, recalling Table 2, DM21@B3LYP performs better on average than B3LYP@B3LYP for TMC117. However, considering the current cost of DM21 (Section II), even a single SCF cycle with DM21 needed for DM21@B3LYP would far exceed the cost of the entire B3LYP@B3LYP calculation.

In summary, we see that DM21 is very effective when it converges, and where it uses already converged B3LYP densities and orbitals. Before concluding, we will attempt to understand why DM21 fails in some cases by examining some of its features, and compare how they differ between cases that converge seamlessly, and those that do not.

## IV. DFT features analysis

To gain insight into DM21's performance in main-group *versus* TMC, in this section we will compare the DM21 features of small molecules. All features in standard hybrid DFAs and the local-hybrid form of DM21 are represented as functions, *f*_a_(**r**), that are defined at some point **r** of interest. Then,1
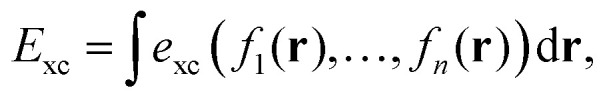
where *n* is the number of features, *f*_1≤*a*≤*n*_(**r**), used to define the local xc energy density, *e*_xc_. For B3LYP there are five ingredients, of which only four are used non-trivially and all are employed analytically – it is thus easy to understand how B3LYP (mis-)behaves. In contrast, understanding how DM21 varies with its *n* = 12 ingredients (*i.e.* dimensions) is a virtually impossible task.

We can, however, get some insights into the kinds of features that DM21 has learned, and those it needs to deal with in systems where it wasn't trained on. Combinations of features that do not appear in the training data are the most likely source of errors in failure cases. For this task, we represent the features of a system using two-dimensional projection heat maps,2
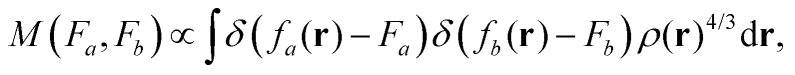
where *f*_*a*_ and *f*_*b*_ are the target features (*e.g. r*_s_^4^|∇*ρ*|) at a given point in space. Data is weighted by the LDA exchange energy density (∝ *ρ*^4/3^) so that the heat map approximates the relative importance of different values of *f*_*a*_ and *f*_*b*_ to the xc energy. Put another way, it represents the likelihood that errors in the DFA at those values will contribute substantially to errors in the xc energy for the system. We focus on features from DM21: the density gradient, |∇*ρ*(**r**)|, kinetic energy density, 
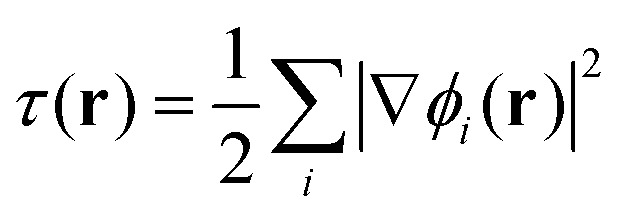
, exchange energy density,3
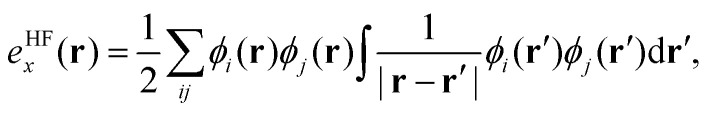
which yields by integration the exact HF exchange energy. Another feature that DM21 uses is the long-range (lr) contribution to *e*^HF^_*x*_(**r**), obtained by splitting the 1/|**r** − **r**′| Coloumb interaction into lr: erf(*ω*|**r** − **r**′|)/|**r** − **r**′| and short-range (sr) part: erfc(*ω*|**r** − **r**′|)/|**r** − **r**′|. In this way, the lr part of *e*^HF^_*x*_(**r**) used by DM21 and is given by:4
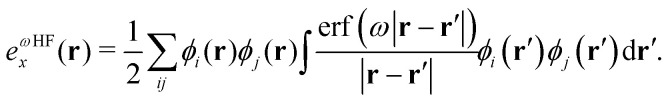
with *ω* set to 0.4 in DM21. The sr part of *e*^HF^_*x*_(**r**) is given by the difference between *e*^HF^_*x*_(**r**) and *e*^*ω*HF^_*x*_(**r**). We make all features unitless by multiplying by powers of the Wigner–Seitz radius, *r*_s_ = 0.62035*ρ*^−1/3^.

**Fig. 7:**
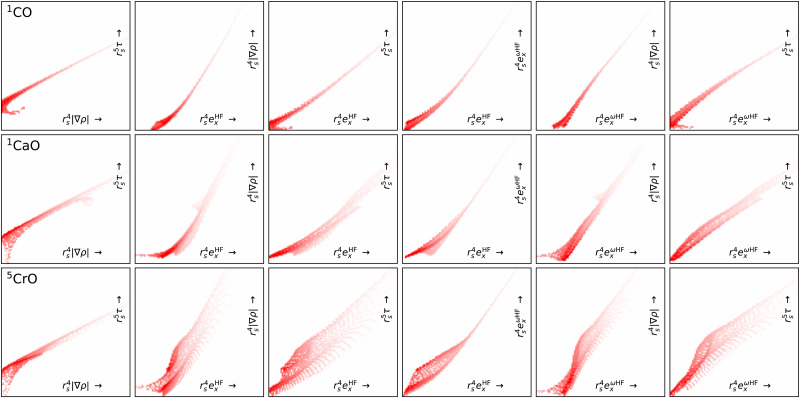
Projection heat maps for different pairs (columns) of unitless features for CO, CaO and CrO (rows). Darker reds indicate more heavily sampled features. White regions indicate a complete absence of features. Dotting indicates incomplete sampling of regions caused by the discrete grid. The bottom and right axes show the features and axes are on a logarithmic scale. We exclude points where *r*_s_ < 1 (*ρ* > 0.24) to remove the nuclear regions from the plots. Data obtained using B3LYP/def2-QZVP.

Fig. 7 shows projection heat maps for six combinations of features for molecular CO, CaO and CrO, all in their lowest energy spin configuration. The features for CO and CaO differ, but in both cases the features are tightly confined to the vicinity of lines. By contrast, CrO has a wider ‘spread’ in feature space, especially as a function of Hartree–Fock exchange energy densities. This means that CrO is more susceptible to errors in the DFA across a wider region of feature space, meaning that a lack of training data in relevant parts of feature space is likely to lead to errors in the DM21 model.

**Fig. 8:**
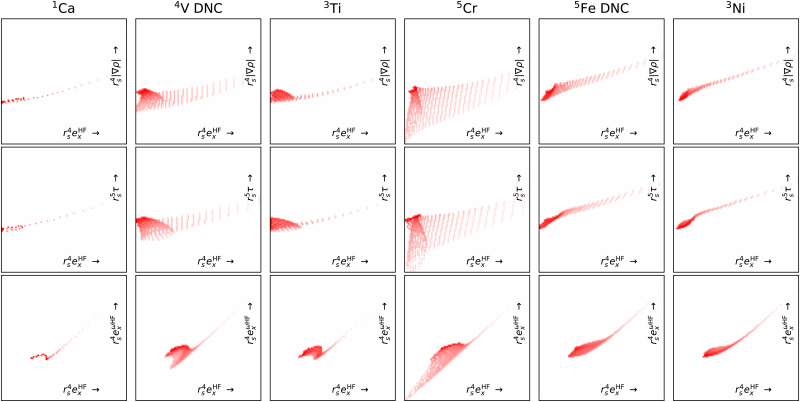
Like Fig. 7 but for atoms (columns) and with fewer features pairs (rows). Note, of these atoms V and Fe did not converge (DNC) using any strategy.

By focusing on atoms, Fig. 8 reveals that the difficulties in CrO are very likely a feature of Cr more than the bond. Indeed, the Cr atom samples a greater spread in feature space than any of the other atoms shown. Given the lack of potential training data even from other transition metals, it is not surprising that DM21 did not learn how to model Cr bonds from its organic training set. What is remarkable is that atomic Cr converges at all, unlike atomic V and Fe that have similar (but less spread) features.

## V. Conclusions and outlook

In conclusion, we have shown that the DM21 functional's performance in transition metal chemistry, despite being comparable in accuracy to B3LYP, faces challenges in SCF convergence that makes it of little to no practical use in this domain. Despite these limitations, we also showed that evaluating DM21 functionals on B3LYP densities results in improved performance over self-consistent B3LYP for TMC117 reactions. To shed light on the SCF convergence issues of DM21 with transition metal molecules, we have analyzed the DM21 features, highlighting the distinctions between transition metal atoms/oxides and their main-group counterparts.

The improved accuracy of DM21@B3LYP over B3LYP@B3LYP demonstrates the significant potential of machine-learned density functionals in transition metal chemistry. Despite its potential, energy refinement on B3LYP densities with DM21 is currently not cost-effective, as a single SCF iteration of DM21 in PySCF for medium-sized molecules can exceed by far the total time required for a B3LYP or even B2PLYP calculation. Carrying out DM21 with B3LYP orbitals seems to offer a useful compromise once DM21 is coupled with a more efficient implementation of the exact exchange energy density.^40^

Moving DFAs beyond the “no man's land” by creating machine-learned functionals with a broad applicability to both main-group and transition metal chemistry remains an open challenge. On the one hand, incorporating features designed to capture strong correlation effects into machine-learning DFAs may improve the transferability to transition metal chemistry.^38,41,42^ On the other hand, addressing this by incorporating transition metal reactions into machine-learning density functionals comes with its own obstacles:

(1) The scarcity of accurate benchmark data for transition metal chemistry is a well-known issue despite recent improvements.^43,44^ For example, the TMC151 database has about ten/thirty times fewer reactions than the GMTKN55/MGCDB84 databases for main-group chemistry.^45,46^ Moreover, within the TMC151 subsets, only TMD60 uses a higher level of theory than CCSD(T), which is a single-reference method. To address this data scarcity, one can either utilize existing^9^ or design new data-efficient strategies for machine-learning DFAs.

(2) Naïvely including transition metal reactions in machine-learning DFAs may compromise the accuracy for main-group chemistry.^14^ However, this can be addressed by employing datasets that are explicitly biased towards ensuring higher transferability to both main-group and transition metal chemistry.^14^

## Conflicts of interest

There are no conflicts to declare.

## Supplementary Material

CP-026-D4CP00878B-s001

## Appendices

### Appendices

#### Appendix A: Strategy D

Strategy D involves a direct orbital minimization algorithm for the orbital-dependent energy, *E*[{*ϕ*_*i*_}], with respect to variations in orbitals within a restricted open-shell theory; with the aim to find (local) minima that are unstable or difficult to find using typical self-consistent field convergence strategies. It involves iteratively solving the approximate-Newton equation, **C** → **C** exp(**A**), where **C** is the matrix describing the orbital coefficients; and **A** is an anti-symmetric matrix with elements,

A1

where,  is a diagonal approximation for the Hessian and *η* = 0.01 is a regularization factor (using occupation factors, *f*_*i*_, and orbital energies, ). For DM21, we can apply the chain-rule to spin-density ingredients to obtain,  where *f*_*iσ*_ indicates whether or not orbital *i* is occupied with spin *σ* in the density; and *F̂*_DM21,*σ*_ is the effective Fock operator for spin *σ*. The optimal solution occurs when ‖**A**‖ → 0 is accompanied by a decrease in energy, indicating that the solution has converged to a minimum.

In fact, Strategy D goes one step further than the direct iteration described above, which helps it to converge difficult cases. After 50 iterations, we set **C** → **C** exp(*α****A**) using an optimal |*α**| < 3. The optimal value, *α**, is determined by quadratically fitting results for *α* ∈ {0,1,2} to find the minimum along the line. This modification helps to avoid rapid variations in energies when outside the radius of convergence for the global minimum and also helps difficult cases iterate to their minimum. Typical calculations (which begin within the radius of convergence) find the minimum within about 30 iterations, so never require this treatment.

The code is available on request.

#### Appendix B: Further computational details

For B3LYP calculations, the convergence threshold is set to 10^−8^, while gradients convergence threshold is set to 10^−4^. In DM21 SCF, they are set to 10^−6^ and 10^−3^, respectively. For DM21 calculations, the orbitals obtained from B3LYP SCF are used as the initial guess. The maximum number of SCF iterations that we use for Strategy A, B and C are set to 200, 500, 900 respectively.
