# Supplementary material for: Deep Mind 21 functional does not extrapolate to transition metal chemistry
Source: Phys Chem Chem Phys. 2024 Apr 4;26(16):12289–98. doi: 10.1039/d4cp00878b (PMC11041869; doi:10.1039/d4cp00878b)
Supplement: CP-026-D4CP00878B-s001 [file CP-026-D4CP00878B-s001.pdf]

Electronic Supplementary Material (ESI) for Physical Chemistry Chemical Physics.
This journal is © the Owner Societies 2024

I. RESULTS ON TMC117 WITHOUT D3(BJ)

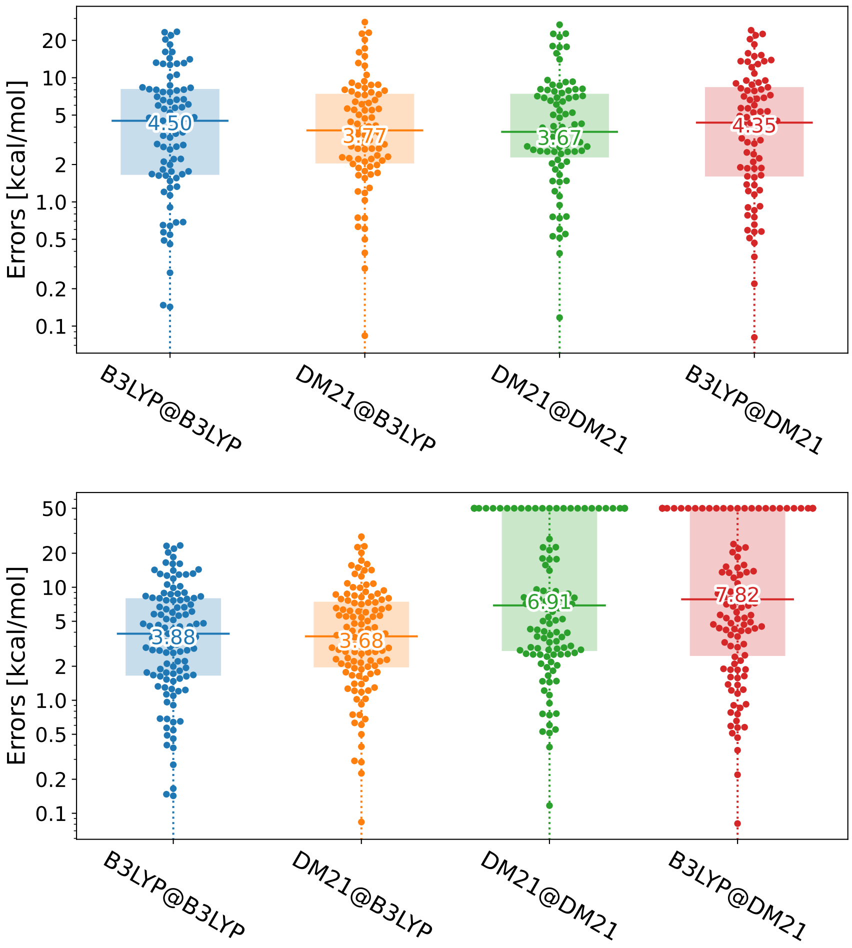

FIG. S1: Beswarm plots same as Fig.1, but without D3(BJ) correction.

dataset	TMB		TMD		MOR
	whole	sub	whole	sub	whole
B3LYP@B3LYP	2.83	2.73	6.34	6.78	9.36
DM21@B3LYP	2.50	2.93	6.67	6.24	8.08
DM21@DM21	-	3.13	-	6.30	8.41
B3LYP@DM21	-	2.66	-	7.11	9.04
Number of Reactions	40	27	60	39	17

TABLE S-I: Same as Tab.II, but without D3(BJ) correction.

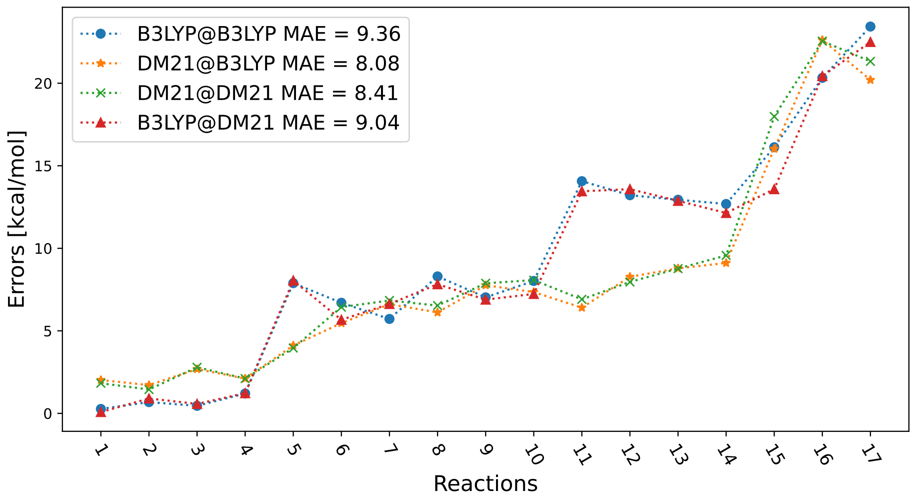

FIG. S2: Same as Fig.4(a), but without D3(BJ) correction.

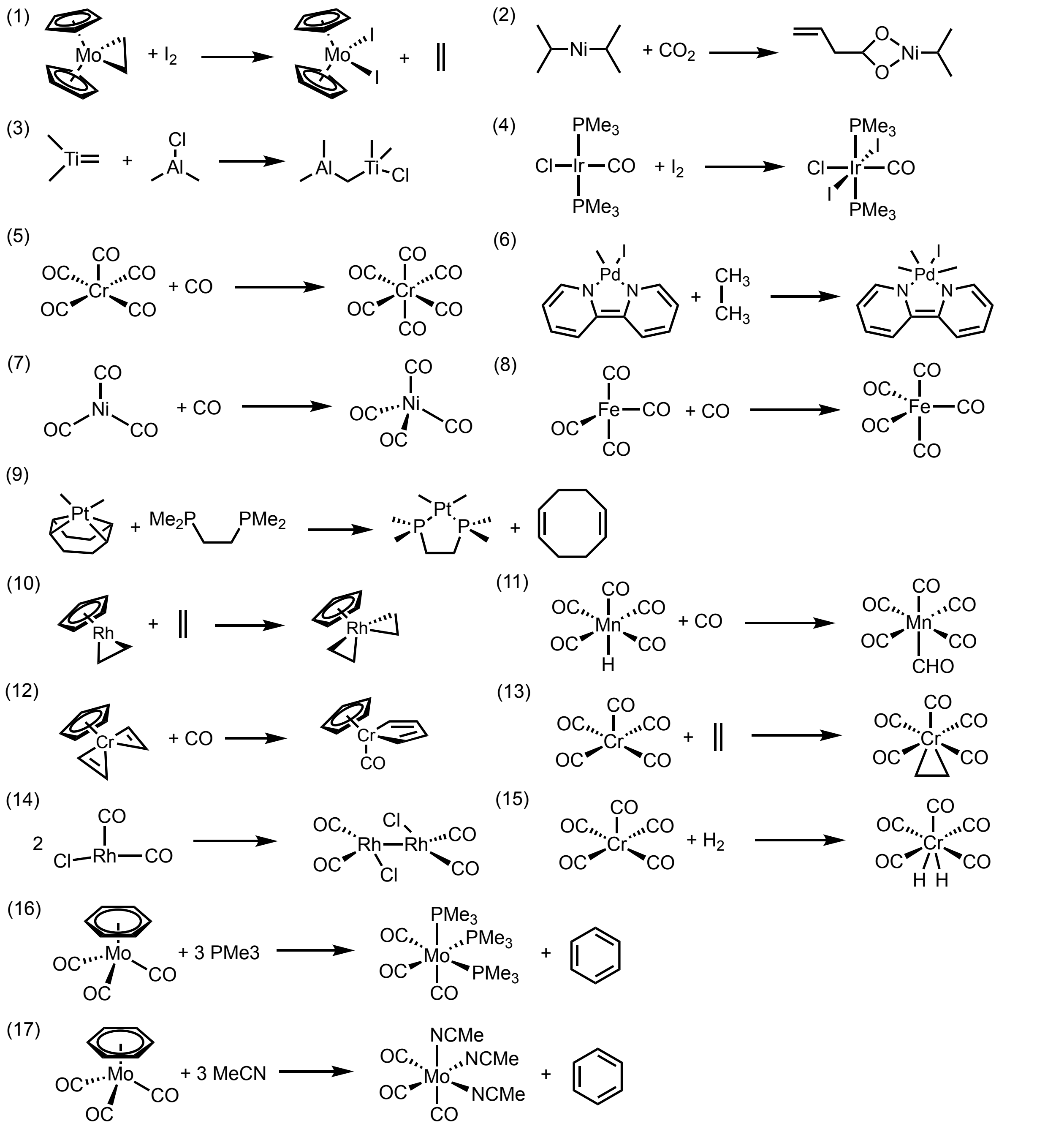

FIG. S3: Reactions contained in MOR17 dataset. The indices correspond to their rankings sorted by the average of D3(BJ) corrected absolute errors from all four methods from small to large, as in Fig. 4(a).

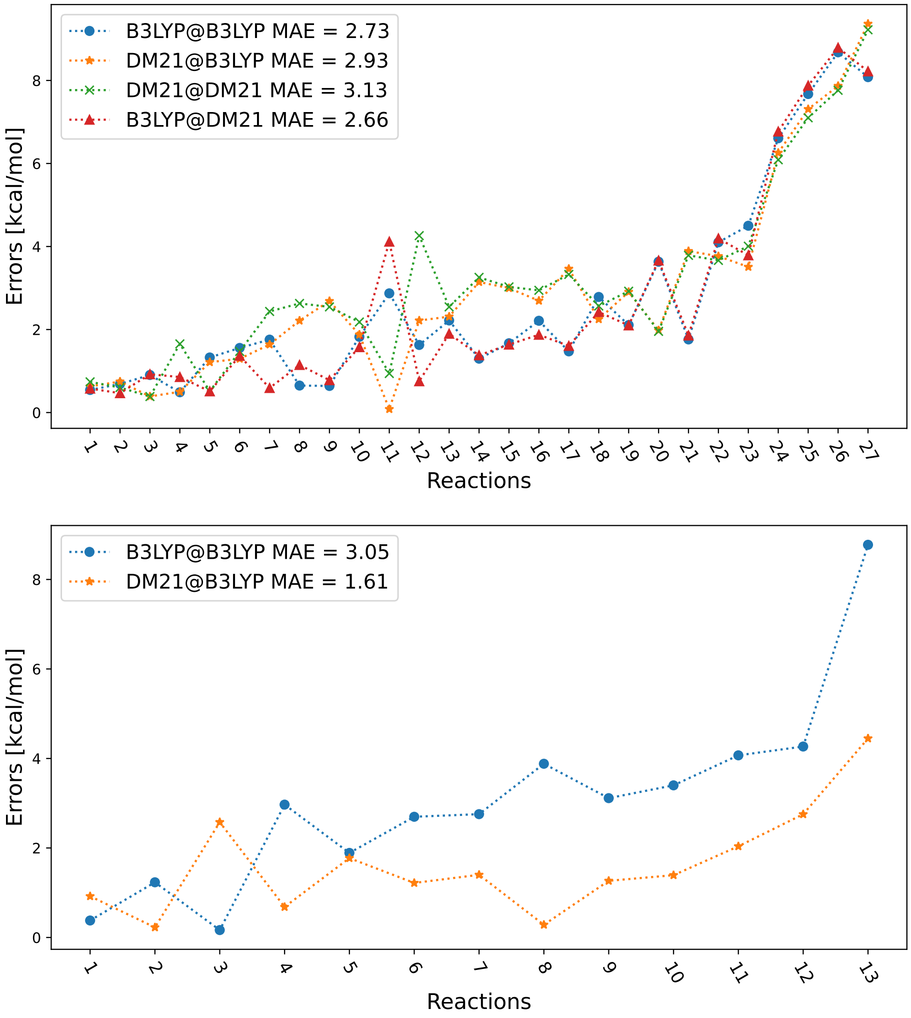

FIG. S4: (a) Same as Fig.5(a), but without D3(BJ) correction. (b) Same as Fig.5(b), but without D3(BJ) correction.

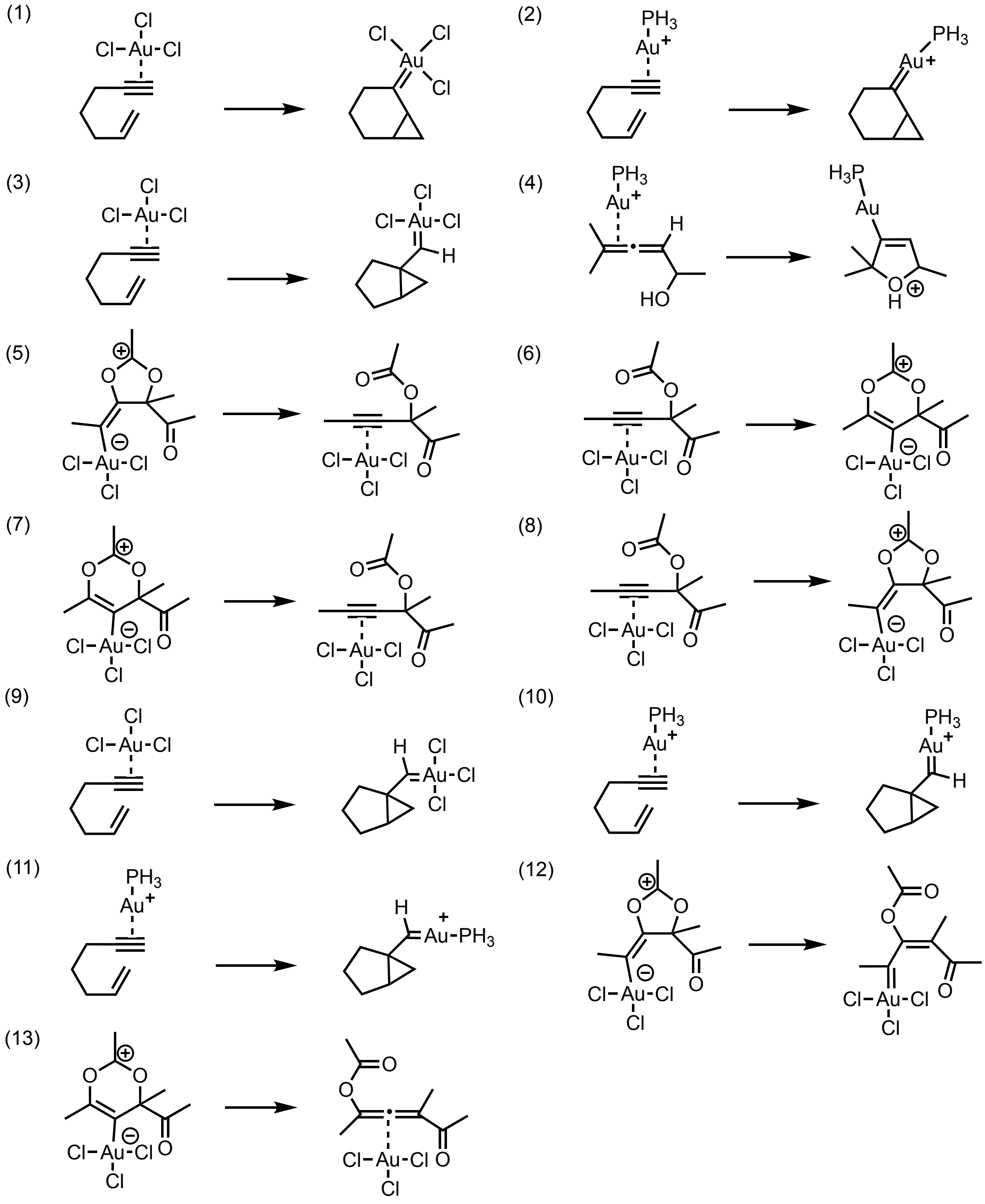

FIG. S5: Reactions contained in TMB40 dataset for which DM21 does not converge. The indices correspond to their rankings sorted by the average of D3(BJ) corrected absolute errors from all four methods from small to large, as in Fig. 5b.

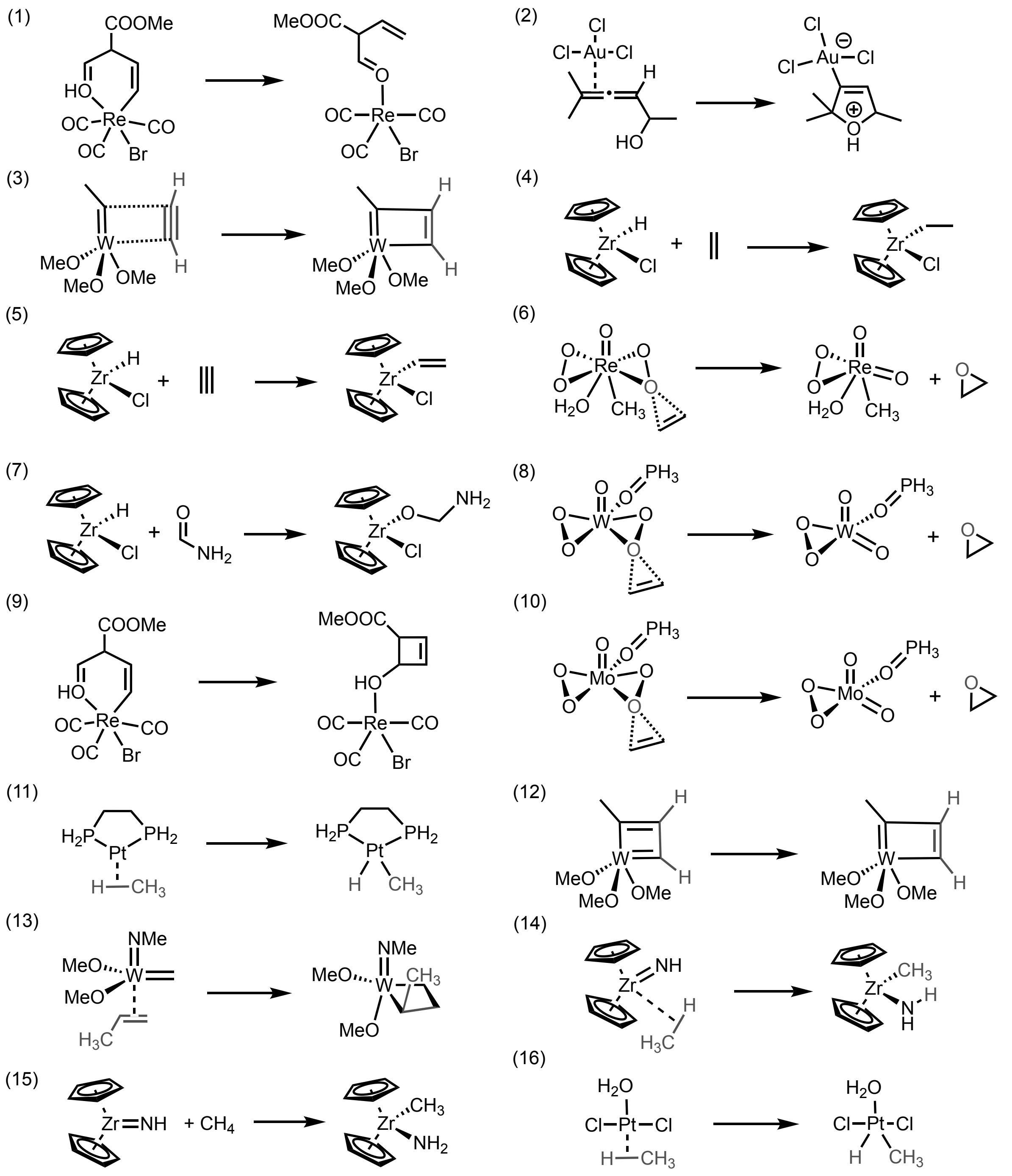

FIG. S6: Reactions contained in TMB40 dataset for which DM21 converges (continued next page).

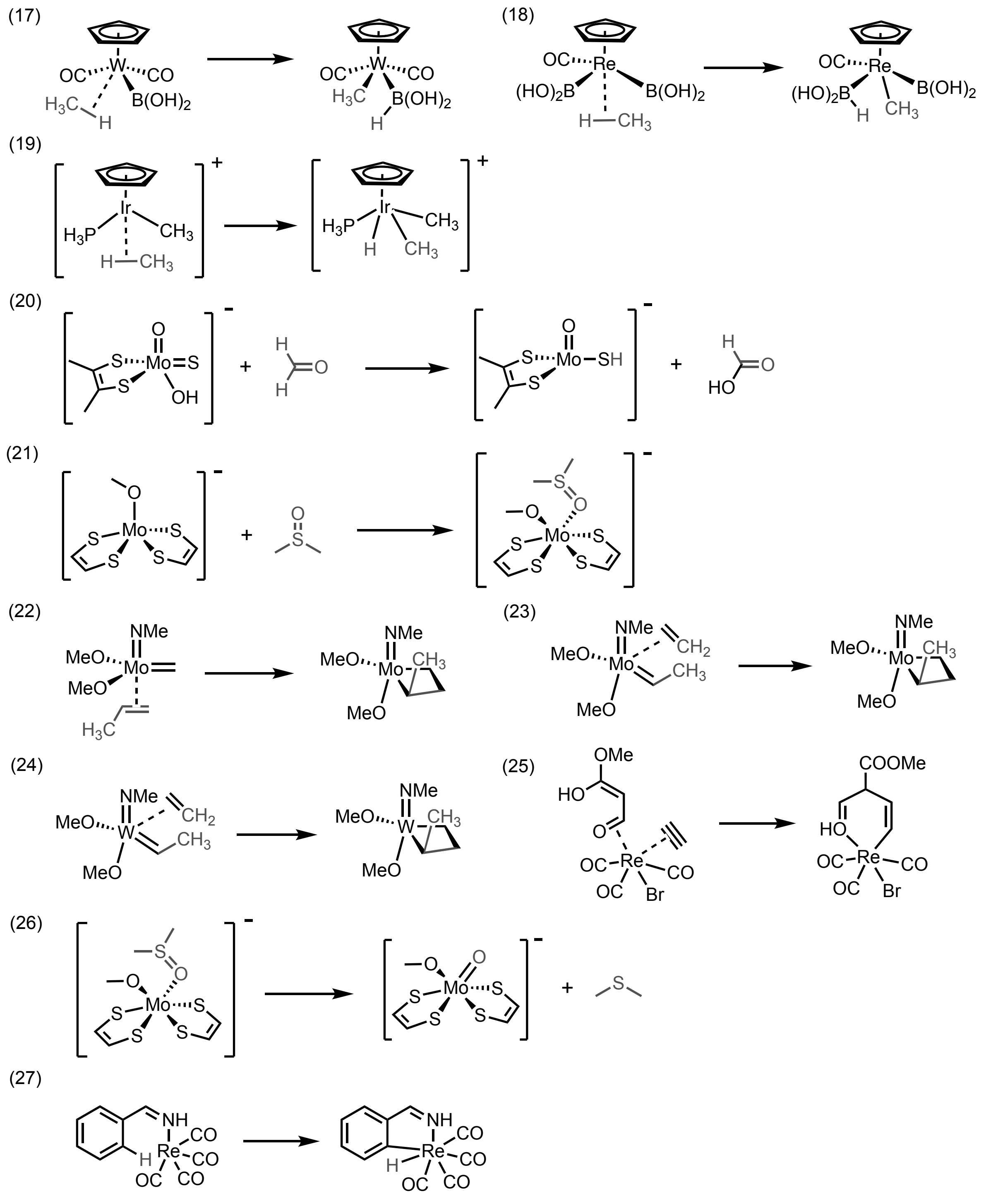

FIG. S7: Reactions contained in TMB40 dataset for which DM21 converges. The indices correspond to their rankings sorted by the average of D3(BJ) corrected absolute errors from all four methods from small to large, as in Fig. 5a.

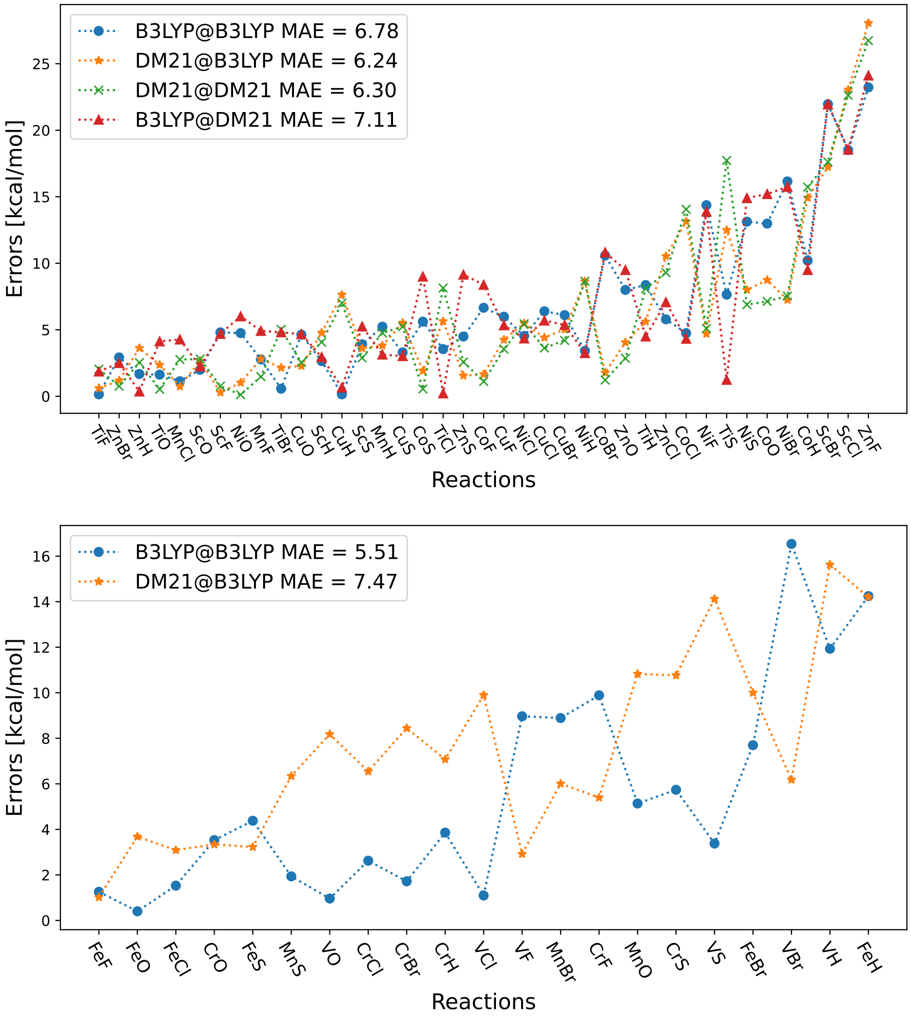

FIG. S8: (a) Same as Tab.6(a), but without D3(BJ) correction. (b) Same as Tab.6(b), but without D3(BJ) correction.

II. CONVERGENCE STATISTICS OF DIFFERENCT STRATEGY

dataset	Atoms				Molecules			
	conv	A	B	C	conv	A	B	C
TMD	14	14	0	0	45	43	2	0
W4-11	12	12	0	0	140	140	0	0

TABLE S-II: DM21 convergence using different strategies
